# Engineering BioBrick vectors from BioBrick parts

**DOI:** 10.1186/1754-1611-2-5

**Published:** 2008-04-14

**Authors:** Reshma P Shetty, Drew Endy, Thomas F Knight

**Affiliations:** 1 Department of Biological Engineering, MIT, 32 Vassar Street Rm 32-311, Cambridge, MA 02139, USA; 2 Department of Biological Engineering, MIT, 77 Massachusetts Avenue Rm 68-580, Cambridge, MA 02139, USA; 3 Computer Science and Artificial Intelligence Laboratory, MIT, 32 Vassar Street Rm 32-312, Cambridge, MA 02139, USA

## Abstract

**Background:**

The underlying goal of synthetic biology is to make the process of engineering biological systems easier. Recent work has focused on defining and developing standard biological parts. The technical standard that has gained the most traction in the synthetic biology community is the BioBrick standard for physical composition of genetic parts. Parts that conform to the BioBrick assembly standard are BioBrick standard biological parts. To date, over 2,000 BioBrick parts have been contributed to, and are available from, the Registry of Standard Biological Parts.

**Results:**

Here we extended the same advantages of BioBrick standard biological parts to the plasmid-based vectors that are used to provide and propagate BioBrick parts. We developed a process for engineering BioBrick vectors from BioBrick parts. We designed a new set of BioBrick parts that encode many useful vector functions. We combined the new parts to make a BioBrick base vector that facilitates BioBrick vector construction. We demonstrated the utility of the process by constructing seven new BioBrick vectors. We also successfully used the resulting vectors to assemble and propagate other BioBrick standard biological parts.

**Conclusion:**

We extended the principles of part reuse and standardization to BioBrick vectors. As a result, myriad new BioBrick vectors can be readily produced from all existing and newly designed BioBrick parts. We invite the synthetic biology community to (1) use the process to make and share new BioBrick vectors; (2) expand the current collection of BioBrick vector parts; and (3) characterize and improve the available collection of BioBrick vector parts.

## Background

The fundamental goal of synthetic biology is to make the process of engineering biology easier. Drawing upon lessons from the invention and development of other fields of engineering, we have been working to produce methods and tools that support the design and construction of genetic systems from standardized biological parts. As developed, collections of standard biological parts will allow biological engineers to assemble many engineered organisms rapidly [1]. For example, individual parts or combinations of parts that encode defined functions (devices) can be independently tested and characterized in order to improve the likelihood that higher-order systems constructed from such devices work as intended (Canton, Labno, and Endy, submitted) [2,3]. As a second example, parts or devices that do not function as expected can be identified, repaired, or replaced readily [4,5].

We define a biological part to be a natural nucleic acid sequence that encodes a definable biological function, and a standard biological part to be a biological part that has been refined in order to conform to one or more defined technical standards. Very little work has been done to standardize the components or processes underlying genetic engineering [6]. For example, in 1996, Rebatchouk *et al*. developed and implemented a general cloning strategy for assembly of nucleic acid fragments [7]. However, the Rebatchouk *et al*. standard for physical composition of biological parts failed to gain widespread acceptance by the biological research community. As a second example, in 1999, Arkin and Endy proposed an initial list of useful standard biological parts but such a collection has not yet been fully realized [8]. In 2003, Knight proposed the BioBrick standard for physical composition of biological parts [9]. Parts that conform to the BioBrick assembly standard are BioBrick standard biological parts. In contrast to the previous two examples, the BioBrick physical composition standard has been used by multiple groups (Canton, Labno, and Endy, submitted) [10-12], and adoption of the standard is growing. For example, each summer, hundreds of students develop and use BioBrick standard biological parts to engineer biological systems of their own design as a part of the International Genetically Engineered Machines competition [13]. Additional technical standards defining BioBrick parts are set via an open standards setting process led by The BioBricks Foundation [14].

The key innovation of the BioBrick assembly standard is that a biological engineer can assemble any two BioBrick parts, and the resulting composite object is itself a BioBrick part that can be combined with any other BioBrick parts. The idempotent physical composition standard underlying BioBrick parts has two fundamental advantages. First, the BioBrick assembly standard enables the distributed production of a collection of compatible biological parts [15]. Two engineers in different parts of the world who have never interacted can each design a part that conforms to the BioBrick assembly standard, and those two parts will be physically composable via the standard. Second, since engineers carry out the exact same operation every time that they want to combine two BioBrick parts, the assembly process is amenable to optimization and automation, in contrast to more traditional *ad hoc *molecular cloning approaches.

The Registry of Standard Biological Parts (Registry) exemplifies the advantage offered by a physical composition standard such as the BioBrick assembly standard [15]. The Registry currently maintains a collection of over 2,000 BioBrick standard biological parts. Every part in the Registry has a BioBrick part number that serves as the unique identifier of the part (for example, BBa_I51020). The Registry maintains information about each part including its sequence, function, and, if available, user experiences. DNA encoding each BioBrick standard biological part is stored and propagated in *Escherichia coli *plasmid-based vectors [16-19]. Biological engineers can obtain parts from the Registry and assemble them using the BioBrick assembly standard in order to construct many-component synthetic biological systems.

All BioBrick parts are currently maintained on a set of plasmids that includes pSB1A3-P1010, pSB3K3-P1010, pSB4A3-P1010 (see Naming of BioBrick vectors in Methods). However, these BioBrick vectors are *ad hoc *designs that were cobbled together from common cloning plasmids such as pUC19 [20-22]. As a result, whenever a new vector is needed for use with BioBrick parts, a biological engineer must design and assemble the new BioBrick vector from scratch.

Several plasmid-based cloning systems that support the manipulation, propagation, and expression of DNA fragments have been developed [20-29]. The Gateway^® ^recombinational cloning system and associated vectors are arguably the closest example of a vector standard in biological research [30,31]. For example, several genome-wide collections of open reading frames (ORFeomes) have been compiled using the Gateway^® ^cloning system [32-34]. The Gateway^® ^system has even been extended to allow assembly of multiple DNA fragments [35,36]. However, the Gateway^® ^system generally requires customized assembly strategies for each new system and therefore does not provide the advantages afforded by the BioBrick standard (above).

Thus, we sought to extend the advantages of BioBrick standard biological parts to the vectors that propagate BioBrick parts. To do this, we developed a new process for engineering BioBrick vectors. The process leverages existing and newly designed BioBrick parts for the ready construction of many BioBrick vectors. To demonstrate the utility of the new process, we constructed seven new BioBrick vectors from the base vector. We also successfully used the new vectors to assemble BioBrick standard biological parts.

## Results

### The BioBrick base vector (BBa_I51020)

**Figure 1:**
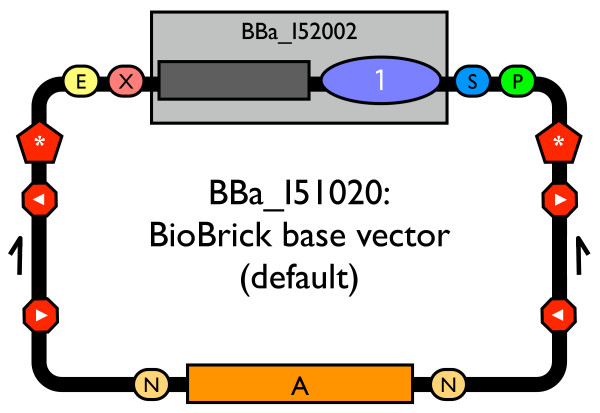
**The BioBrick base vector (BBa_I51020)**. Schematic diagram of BBa_I51020: a BioBrick base vector designed to facilitate construction of new BioBrick vectors. Parts from the collection listed in Figure 5 were used to construct BBa_I51020.

The process for engineering BioBrick vectors from BioBrick parts is primarily based upon a newly designed BioBrick part: BBa_I51020 [Genbank:EU496089]. The new part is a BioBrick base vector that serves as a scaffold for construction of new BioBrick vectors (Figure 1). Starting from the base vector, new vectors can be built using plasmid replication origins and antibiotic resistance markers that conform to the BioBrick standard for physical composition. Thus, the base vector enables the ready reuse of vector parts available from the Registry of Standard Biological Parts. Use of the base vector to construct BioBrick vectors ensures standardization and uniformity in any resulting BioBrick vectors. For convenience, the base vector includes both a high copy replication origin and ampicillin resistance marker, so the base vector itself is capable of autonomous plasmid replication for easy DNA propagation and purification [37].

All BioBrick vectors derived from the BioBrick base vector have five key features. First, BioBrick vectors include a complete BioBrick cloning site to support the propagation and assembly of BioBrick standard biological parts [9]. Second, BioBrick vectors contain a positive selection marker in the cloning site to ameliorate one of the most common problems during assembly of BioBrick parts: contamination of the ligation reaction with uncut plasmid DNA [38]. Any cells transformed with the BioBrick vector produce the toxic protein CcdB and do not grow [39-41]. Cloning a BioBrick part into the cloning site of the vector removes the toxic *ccdB *gene. Third, BioBrick vectors contain a high copy origin in the cloning site to facilitate increased yields from plasmid DNA purification [42,43]. Again, cloning a BioBrick part into the cloning site removes the high copy origin in the cloning site thereby restoring replication control to the vector origin. Fourth, BioBrick vectors include transcriptional terminators and translational stop codons flanking the cloning site to insulate the proper maintenance and propagation of the vector from any possibly disruptive function encoded by inserted BioBrick parts [44-47]. Fifth, BioBrick vectors include verification primer annealing sites sufficiently distant from the cloning site to check the length and sequence of the cloned BioBrick part. The primer annealing sites are identical to those found in commonly used BioBrick vectors, such as pSB1A3-P1010, to support backwards compatibility.

### Constructing new BioBrick vectors using the BioBrick base vector

**Figure 2:**
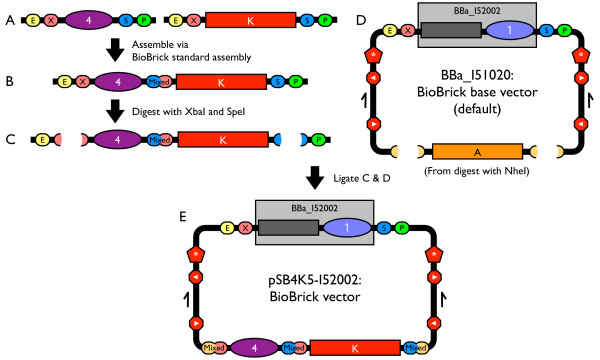
**How to build new BioBrick vectors**. Assembly strategy for a new BioBrick vector using the BioBrick base vector BBa_I51020. (A) The replication origin and antibiotic resistance cassette should each be BioBrick standard biological parts. (B) Assemble the desired replication origin and antibiotic resistance cassette via BioBrick standard assembly to construct a composite origin and antibiotic resistance cassette. (C) Digest the resulting BioBrick composite part with XbaI and SpeI. (D) To excise the ampicillin resistance marker, digest the base vector with NheI. XbaI, SpeI, and NheI all generate compatible cohesive DNA ends that, when ligated with a DNA end from a one of the other enzymes, produce a non-palindromic sequence that cannot be cut by any of the three enzymes. Finally, ligate the digested composite origin and resistance marker to the digested base vector. (E) The result is the new BioBrick vector pSB4K5-I52002.

Constructing new BioBrick vectors starting from the BioBrick base vector requires just two assembly steps (Figure 2). The replication origin and antibiotic resistance marker should each be BioBrick standard parts. To construct a BioBrick vector, assemble the origin and antibiotic resistance marker via BioBrick standard assembly (first assembly step). Then, digest the resulting composite part with restriction enzymes XbaI and SpeI, and digest the BioBrick base vector with NheI to excise the ampicillin resistance marker. Next, ligate the composite origin and resistance marker to the linearized base vector (second assembly step). XbaI, SpeI, and NheI all generate compatible DNA ends that, when ligated with a DNA end from one of the other enzymes, produce a non-palindromic sequence that cannot be cut by any of the three enzymes. Thus, proper assembly of the vector eliminates any BioBrick enzyme sites and ensures that the resulting vector adheres to the BioBrick physical composition standard. Finally, transform the ligation product into a strain tolerant of *ccdB *expression, such as *E. coli *strain DB3.1 [48,49].

To support the construction of new BioBrick vectors, we built four new antibiotic resistance markers and two replication origins all as BioBrick standard biological parts. The four antibiotic resistance markers express proteins that confer resistance to ampicillin (BBa_P1002 [Genbank:EU496092]), kanamycin (BBa_P1003 [Genbank:EU496093]), chloramphenicol (BBa_P1004 [Genbank:EU496094]), and tetracycline (BBa_P1005 [Genbank:EU496095]), respectively [50-53]. The two replication origins were derived from the pSC101 (BBa_I50042 [Genbank:EU496096]) and p15A (BBa_I50032 [Genbank:EU496097]) replicons, respectively [54,55]. We used the described procedure, base vector, and new vector parts to construct seven new BioBrick vectors: pSB4A5-I52002, pSB4K5-I52002, pSB4C5-I52002, pSB4T5-I52001, pSB3K5-I52002, pSB3C5-I52001, and pSB3T5-I52001 [Genbank:EU496098–EU496104].

### Assembling BioBrick parts using a new BioBrick vector

**Figure 3:**
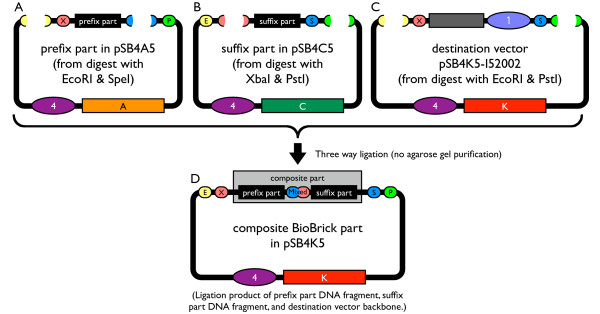
**How to use a new BioBrick vector for standard assembly**. Assembly strategy for two BioBrick standard biological parts using a new BioBrick vector. (A) Digest the prefix part with enzymes EcoRI and SpeI. (B) Digest the suffix part with restriction enzymes XbaI and PstI. (C) Digest the destination vector (pSB4K5-I52002) into which the two parts will be assembled with restriction enzymes EcoRI and PstI. Without agarose gel purification of the linearized DNA, ligate the three fragments, transform into *E. coli *and plate on LB agar plates supplemented with the antibiotic corresponding to the destination vector resistance marker. (D) Most of the resulting colonies contain the composite BioBrick part cloned into the destination vector.

BioBrick vectors support assembly of new BioBrick standard parts. The new vectors are compatible with prefix or postfix insertions of BioBrick parts as originally described [9]. Alternatively, the new vectors also support three antibiotic based assembly (3A assembly; Figure 3; Shetty, Rettberg, and Knight, in preparation) [56]. 3A assembly is a method for assembling one part (the prefix part) upstream or 5' to a second part (the suffix part) in the BioBrick cloning site of a BioBrick vector (the destination vector). 3A assembly favors correct assembly of the prefix and suffix BioBrick parts in the destination vector through a combination of positive and negative selection. Briefly, 3A assembly works as follows: Digest the prefix part with EcoRI and SpeI, the suffix part with XbaI and PstI, and the destination vector with EcoRI and PstI. Then, ligate the two parts and destination vector and transform into competent *E. coli*. Plate the tranformed cells on LB agar plates supplemented with antibiotic corresponding to the destination vector resistance marker. Most of the resulting colonies should contain the composite BioBrick part cloned into the destination vector.

**Figure 4:**
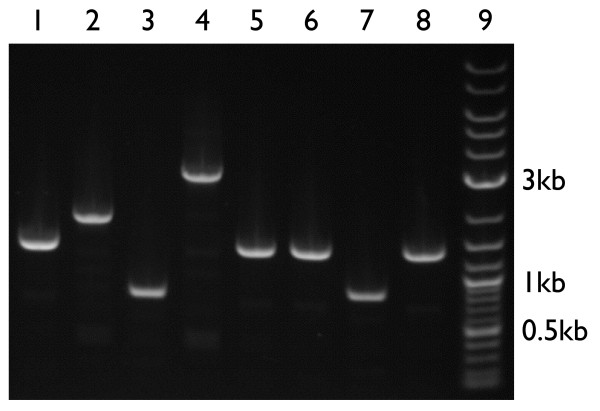
**Using the new BioBrick vectors**. To verify the function of the new BioBrick vectors, we performed a colony PCR using primers that anneal to the verification primer binding sites. To check the length of the resulting PCR products, we electrophoresed the reactions through an 0.8% agarose gel. Lanes 1–8 are the PCR products resulting from the amplification of the following BioBrick parts cloned into new BioBrick vectors. The desired PCR product lengths are in parentheses. Lane 1 is pSB4A5-I52001 (1370 bp), lane 2 is pSB4K5-T9003 (1883 bp), lane 3 is pSB4C5-E0435 (814 bp), lane 4 is pSB4T5-P20061 (2988 bp), lane 5 is pSB3K5-I52002 (1370 bp), lane 6 is pSB3C5-I52001 (1370 bp), lane 7 is pSB3T5-I6413 (867 bp), and lane 8 is BBa_I51020 (1370 bp). Lane 9 is 1 *μ*g of 2-log DNA ladder (New England Biolabs, Inc.). The 0.5 kb, 1 kb, and 3 kb DNA fragments in the DNA ladder are annotated.

To confirm that our new BioBrick vectors function as expected, we assembled new BioBrick standard biological parts using four of the vectors that we constructed. To demonstrate that the composite BioBrick parts were correctly assembled using our new vectors, we performed a colony PCR amplification of the assembled parts and determined that the PCR product length was correct (Figure 4). Each part was also verified to be correct via sequencing with primers that anneal to the verification primer binding sites (BBa_G00100 and BBa_G00102).

## Discussion

We developed a new process for engineering BioBrick vectors from BioBrick parts. The process now makes possible the ready construction of many, new BioBrick vectors using the growing collection of BioBrick parts available from the Registry of Standard Biological Parts. Moreover, new BioBrick vectors can be constructed from the BioBrick base vector in just two assembly steps. Finally, any BioBrick vectors derived from the BioBrick base vector have five key features designed to facilitate the cloning, assembly, and propagation of BioBrick parts. We used the process to construct seven new BioBrick vectors and used the vectors to assemble new BioBrick parts.

### Design of new BioBrick vectors parts

**Figure 5:**
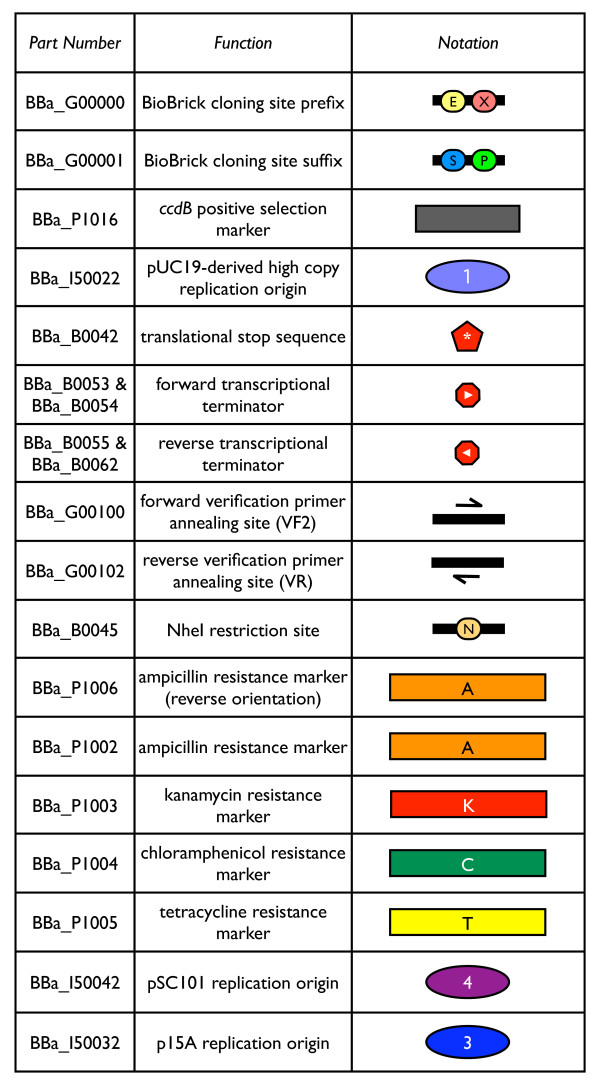
**New BioBrick vector parts**. The Registry part number, function, and graphical notation of each constructed BioBrick vector part are listed. The part collection includes (1) BBa_G00000: BioBrick cloning site prefix including the EcoRI (E) and XbaI (X) restriction enzyme sites, (2) BBa_G00001: BioBrick cloning site suffix including the SpeI (S) and PstI (P) restriction enzyme sites which, together with the BioBrick prefix, forms a BioBrick cloning site for compatibility with all BioBrick standard biological parts, (3) BBa_P1016: positive selection marker *ccdB *to improve yield of insert-containing clones during part assemblies, (4) BBa_I50022: pUC19-derived high copy replication origin within the BioBrick cloning site that allows for easy plasmid DNA purification of the base vector and any derived vectors, (5) BBa_B0042: a short DNA sequence that has translational stop codons in all six reading frames to prevent translation into or out of the BioBrick cloning site, (6) BBa_B0053-B0055 and BBa_B0062: forward and reverse transcriptional terminators flanking the BioBrick cloning site to prevent transcription into or out of the BioBrick cloning site, (7) BBa_G00100 and BBa_G00102: sequence verification primer annealing sites for primers VF2 and VR, (8) BBa_B0045: NheI (N) restriction site for insertion of desired replication origin and resistance marker to construct vector of interest, (9) BBa_P1006: ampicillin resistance selection marker to facilitate propagation of the base vector, (10) BBa_P1002-P1005: four antibiotic resistance markers, and (11) BBa_I50042 and BBa_I50032: pSC101 and p15A replication origins. Each part is used either as a component of the BioBrick base vector BBa_I51020 (1–9) or to construct new BioBrick vectors (10–11).

To adhere to the BioBrick standard for physical composition, BioBrick vector parts need only be free of the BioBrick restriction enzyme sites. However, we chose to design anew all BioBrick vector parts (Figure 5), so that we could completely specify their DNA sequences. We compiled a list of potentially useful endonuclease sites for removal from all new BioBrick vector parts (Table 1). We targeted each group of endonuclease sites for removal for a different reason. We targeted recognition sites of enzymes that produce compatible cohesive ends to the BioBrick enzymes because such enzymes often prove useful in constructing new variants of BioBrick vectors. We targeted offset cutter sites because they may be useful in alternative restriction enzyme-based assembly methods [57]. We targeted homing endonuclease sites because they are commonly used in genome engineering [58]. We targeted some nicking endonuclease sites because they can be useful for specialized cloning applications [59]. Finally, we targeted several additional restriction endonuclease sites to keep them available for use by new standards for physical composition. Our list of endonuclease sites constitutes a set of target sequences that should be considered for removal from any newly synthesized BioBrick part, if possible. The target sequence set will change as the synthetic biology community develops new standards for physical composition of BioBrick parts. Some of the targeted endonuclease sites were naturally absent from the DNA sequences encoding our new vector parts. For any remaining sites, we removed the recognition sequences from the BioBrick vector parts by introducing point mutations. However, the functions of the pSC101 and pUC19-derived plasmid replication origins were sensitive to introduced mutations, so the replication origins used in this work are not free of all targeted endonuclease sites (see Methods). Similarly, issues during synthesis led to an unnecessary redesign of the *ccdB *positive selection marker, so it too is not free of all targeted endonuclease sites.

**Table 1:** Endonuclease sites targeted for removal from BioBrick vector parts.

*Endonuclease*	*Description*
EcoRI, XbaI, SpeI, PstI	BioBrick restriction site
ApoI, MfeI	Produces compatible ends to EcoRI
AvrII, NheI	Produces compatible ends to XbaI and SpeI
NsiI SbfI	Produces compatible ends to PstI
AarI, AcuI, BbsI, BciVI, BfuAI, BmrI, BsaI, BsgI, BsmBI, BsmI, BspMI, BsrDI, BtgZI, EarI, EcoP15I, FokI, SapI, TspRI	Offset cutter
I-CeuI, I-SceI, PI-PspI, PI-SceI, I-PpoI	Homing endonuclease
Nt.BbvCI, Nt.BstNBI, Nt.AlwI	Nicking endonuclease
AgeI, AscI, BamHI, BbvCI, FseI, HindIII, KasI, NcoI, NdeI, NgoMIV, PacI, PmeI (MssI), RsrII, SacI, SalI, SfiI, SgfI, SgrAI, SrfI, SwaI (SmiI), XcmI, XhoI, XmaI, XmnI, ZraI	Restriction endonuclease

A list of endonuclease sites targeted for removal from BioBrick vectors parts. The endonuclease sites were targeted for removal to enable various end-user DNA cloning and manipulation applications.

### Construction of BioBrick base vector

To realize our designs for new BioBrick vectors, we contracted for DNA synthesis of the four antibiotic resistance markers, pSC101 replication origin and the entire BioBrick base vector. However, synthesis of the BioBrick base vector was problematic (see Methods). The issues that arose during synthesis are briefly discussed here, because they are relevant to anyone interested in synthesizing new BioBrick parts. Difficulties during synthesis stemmed from the inclusion of both a *ccdB *positive selection marker that is toxic to most *E. coli *strains and a synthetic replication origin that proved incapable of supporting replication of the BioBrick base vector. Commercial DNA synthesis processes currently rely on cloning, assembly, and propagation of synthesized DNA in *E. coli*. In general, for parts whose function are incompatible with growth and replication of *E. coli*, the processes of DNA design and DNA synthesis cannot be easily decoupled. Improvements in commercial DNA synthesis are needed that free the process from dependence on *in vivo *DNA propagation and replication.

## Conclusion

The goal of synthetic biology is to make the process of design and construction of many-component, engineered biological systems easier. In support of this goal, a technical standard for the physical composition of biological parts was developed [9]. Here, we extended the same principles of part reusability and standardization of physical composition to the vectors that are used to assemble and propagate BioBrick parts. Using the process described here, new BioBrick vectors can be produced from existing and newly designed BioBrick parts. As a result, myriad new vectors with diverse functions can be built readily to support the engineering of many-component systems. We invite the community to build on this work in several ways. First, we invite the community to use the process described here to construct more BioBrick vectors and share them via the Registry of Standard Biological Parts. Second, we invite the community to expand the collection of parts for making BioBrick vectors. For example, shuttle vector parts, compatible replication origins, and additional antibiotic resistance markers would all be useful contributions to the Registry. Third, we invite the community to further characterize and improve the BioBrick parts that make up BioBrick vectors. For example, important parameters to measure include plasmid copy number, and transcriptional and translational read-through into and out of the BioBrick cloning site.

## Methods

### Design of BioBrick vector parts and the BioBrick base vector

We designed all BioBrick vector parts and the BioBrick base vector using Vector NTI^® ^Suite 7 for Mac OS X by Invitrogen Life Science Software in Carlsbad, CA. We removed endonuclease recognition sites from the designed parts either manually or using GeneDesign v*β;*2.1 Rev 5/26/06 [60].

### Construction of BioBrick vector parts

We contracted for DNA synthesis of the four antibiotic resistance markers and the pSC101 replication origin to the DNA synthesis company Codon Devices, Inc. in Cambridge, MA. The four antibiotic resistance markers (BBa_P1002-P1005) were easily synthesized as designed. Testing confirmed that the four markers conferred resistance to the corresponding antibiotics. Synthesis of the pSC101 origin was also straightforward. However, testing revealed that our design for the pSC101 origin (BBa_I50040) was nonfunctional as a replication origin. We successfully reconstructed a functional pSC101 replication origin (BBa_I50042) via PCR of an existing plasmid. Thus, we presume that one or more of the introduced point mutations to eliminate endonuclease sites were deleterious to the plasmid replication function of the designed origin. We did not attempt to synthesize the p15A replication origin (BBa_I50032). Instead, like the pSC101 origin, we constructed p15A origin by PCR of an existing plasmid.

We constructed the functional pSC101 replication origin by PCR using pSB4A3-P1010 as a template and amplification primers I50042-f (5'-GTT TCT TCG AAT TCG CGG CCG CTT CTA GAG CTG TCA GAC CAA GTT TAC GAG-3') and I50042-r (5'-GTT TCT TCC TGC AGC GGC CGC TAC TAG TAG TTA CAT TGT CGA TCT GTT C-3'). We constructed the p15A replication origin by PCR using pSB3K3-P1010 as a template and amplification primers I50032-f (5'-GTT TCT TCG AAT TCG CGG CCG CTT CTA GAG ATG GAA TAG ACT GGA TGG AG-3') and I50032-r (5'-GTT TCT TCC TGC AGC GGC CGC TAC TAG TAA ACA CCC CTT GTA TTA CTG-3'). Each reaction was a mix of 45 *μ*L PCR SuperMix High Fidelity, 31.25 pmoles each of forward and reverse primer, and 1 ng template DNA in a 50 *μ*L total volume. The PCR conditions were an initial denaturation step of 95°C for 15 mins followed by 40 cycles of 94°C for 30 seconds, 56°C for 30 seconds, and 68°C for 2.5 minutes. Finally, the reactions were incubated at 68°C for 20 minutes. We then added 20 units DpnI restriction enzyme to each reaction to digest the template DNA. The reactions were incubated for 2 hours at 37°C and then heat-inactivated for 20 minutes at 80°C. We purified both reactions using a MinElute PCR Purification kit according to the manufacturer's directions (QIAGEN, Germany). The pSC101 and p15A origin PCR products were used directly for assembly of the BioBrick vectors.

### Construction of BioBrick base vector

We also contracted for synthesis of the entire BioBrick base vector. However, we encountered two issues during synthesis of the base vector. First, troubleshooting efforts during synthesis compromised the design of the base vector: failed attempts to clone the base vector into an *E. coli *strain intolerant of expression of the toxic protein CcdB led to an unnecessary redesign of the *ccdB *positive selection marker in the BioBrick base vector (from BBa_P1011 to BBa_P1016 [Genbank:EU496090]). Second, faulty part design adversely impacted the synthesis process: our pUC19-based replication origin design was similarly nonfunctional, so the base vector could not be propagated as specified. Yet, synthesized DNA for the BioBrick base vector was nevertheless provided. We eventually determined that the provided DNA was actually a fusion of two slightly different copies of the base vector: one with the designed, nonfunctional version of the pUC19 origin (BBa_I50020) and one with a functional version of the pUC19 origin (BBa_I50022 [Genbank:EU496091]). To obtain a single, corrected version of the BioBrick base vector, we performed a restriction digest of the provided base vector DNA with EcoRI. We then re-ligated 1 *μ*L of a ten-fold dilution of the linearized base vector DNA. For detailed reaction conditions, see Assembly of BioBrick parts using the new BioBrick vectors. We transformed the religated BioBrick base vector into *E. coli *strain DB3.1 via electroporation and plated the transformed cells on LB agar plates supplemented with 100 *μ*g/mL ampicillin to obtain the corrected BioBrick base vector BBa_I51020 [48,61,62]. Correct construction of the BioBrick base vector was verified by DNA sequencing by the MIT Biopolymers Laboratory.

### Assembly of BioBrick vectors

We assembled the new BioBrick vectors as described (Figure 2). For detailed reaction conditions, see Assembly of BioBrick parts using the new BioBrick vectors. However, we used the synthesized BioBrick base vector BBa_I51019 instead of the corrected BioBrick base vector BBa_I51020, since, at the time, we had not yet identified the issue with the provided synthesized DNA. As a result, we obtained a mixture of new vectors. Four of the constructed vectors have a functional version of the pUC19 origin (BBa_I50022) in the BioBrick cloning site and propagate at high copy (vectors with BBa_I52002: pSB4A5, pSB4K5, pSB4C5, and pSB3K5). The other three vectors have a nonfunctional version of the pUC19 origin (BBa_I50020) in the BioBrick cloning site and propagate at low copy (vectors with BBa_I52001: pSB4T5, pSB3C5, and pSB3T5). We chose to describe all seven vectors here for two reasons. First, all seven new BioBrick vectors can be used for the propagation and assembly of BioBrick parts; the vectors pSB4T5, pSB3C5, and pSB3T5 are just slightly less convenient for plasmid DNA purification. Second, the difficulties that we encountered during construction of the BioBrick base vector are illustrative of the current interdependence of DNA design and DNA synthesis (see Discussion).

### Assembly of BioBrick parts using the new BioBrick vectors

We assembled BioBrick composite parts as described (Figure 3). We performed all restriction digests by mixing 0.5–1 *μ*g DNA, 1X NEBuffer 2, 100 *μ*g/ml Bovine Serum Albumin, and 1 *μ*L each needed restriction enzyme in a 50 *μ*L total volume. Restriction digest reactions were incubated for at least 2 hours at 37°C and then heat-inactivated for 20 minutes at 80°C. We then dephosphorylated the destination vector into which the parts were assembled. (When assembling BioBrick vectors, we dephosphorylated the composite origin and resistance marker to prevent circularization of this DNA fragment.) We performed dephosphorylation reactions by adding 5 units Antarctic Phosphatase and 1X Antarctic Phosphatase Reaction Buffer in a total volume of 60 *μ*L to the heat-inactivated restriction digest reaction. We incubated dephosphorylation reactions for 1 hour at 37°C and inactivated the phosphatase by heating to 65°C for 5 minutes. We purified all reactions using a MinElute PCR Purification kit according to the manufacturer's directions (QIAGEN). We performed all ligation steps by mixing 2–4 *μ*L of each purified, linearized DNA, 1X T4 DNA Ligase Reaction Buffer, and 200 units T4 DNA Ligase in a 10 *μ*L total volume. We incubated the ligation reactions for 20 minutes at room temperature. We transformed all assembled BioBrick parts into *E. coli *strain TOP10 via chemical transformation [63-65]. (We transformed the assembled BioBrick vectors into *E. coli *strain DB3.1 via electroporation [48,61,62].) Transformed cells were plated on LB agar plates supplemented with 100 *μ*g/mL ampicillin, 50 *μ*g/mL kanamycin, 35 *μ*g/mL chloramphenicol, or 15 *μ*g/mL tetracycline as appropriate. We identified clones with correct construction of BioBrick parts by growth on the plates supplemented with the correct antibiotic, lack of growth on plates supplemented with other antibiotics, length verification by colony PCR (see next section), and DNA sequencing by the MIT Biopolymers Laboratory.

### Verification of correct BioBrick part assembly via colony PCR

To demonstrate the correct assembly of BioBrick parts using the new BioBrick vectors, we performed a colony PCR using primers that anneal to the verification primer binding sites. We picked one colony and diluted it into 100 *μ*L water. Then we mixed 9 *μ*L PCR SuperMix High Fidelity, 6.25 pmoles VF2 primer (5'-TGC CAC CTG ACG TCT AAG AA-3'), 6.25 pmoles VR primer (5'-ATT ACC GCC TTT GAG TGA GC-3'), and 1 *μ*L colony suspension. The PCR conditions were as described previously but using an annealing temperature of 62°C and an elongation time of 3.5 minutes. We diluted the reactions four-fold with water and then performed an agarose gel electrophoresis of 20 *μ*L of each diluted reaction using a 0.8% E-Gel^®^. We also electrophoresed 1 *μ*g of 2-log DNA ladder (New England Biolabs, Inc., Ipswich, MA) to verify the length of each PCR product. The gel was imaged with 302 nm transilluminating ultraviolet light using an ethidium bromide emission filter and an exposure time of 614 milliseconds.

Materials for all PCR and agarose gel electrophoresis steps in this work were purchased from the Invitrogen Corporation in Carlsbad, CA unless otherwise specified. Reagents for all restriction digest, dephophorylation, and ligation reactions were purchased from New England Biolabs, Inc., Ipswich, MA. All PCR and temperature-controlled incubation steps were done in a DNA Engine Peltier Thermal Cycler (PTC-200) or DNA Engine OPTICON™from MJ Research, Inc. (now Bio-Rad Laboratories, Inc., Hercules, CA).

### Naming of BioBrick vectors

BioBrick vector names take the form pSB#X#. The letters pSB are an acronym for plasmid Synthetic Biology. The first number denotes the origin of replication (Table 2). The letter X identifies the antibiotic resistance marker(s) present in the vector (Table 3). Vectors with multiple resistance markers have multiple, successive letters. Finally, the last number in the vector name is a version number to differentiate between the various implementations of the pSB series of vectors (Table 4). When referring to both a BioBrick standard biological part and the vector in which it is cloned, the convention is to use the form [vector name]- [part number] such as pSB4K5-T9003. To refer to BioBrick vectors to be used for construction of BioBrick parts, use the full vector name and default cloned part. For example, pSB4A3-P1010, pSB1A10-P1010, pSB4K5-I52002, and pSB3T5-I52001 are all available vectors from the Registry of Standard Biological Parts. However, for convenience, vector names are often abbreviated to pSB4A3, pSB1A10, pSB4K5, and pSB3T5, respectively. New plasmid-based vectors constructed from the BioBrick base vector BBa_I51020 should be named pSB#X5-I52002 where the # is determined by the identity of the replication origin and the letter X is determined by the antibiotic resistance marker(s) present. To expand the BioBrick vector nomenclature, submit new vectors or vector parts to the Registry of Standard Biological Parts and document any new annotation needed [66]. The BioBricks Foundation is leading an open standards setting process should any revisions to the BioBrick vector nomenclature beyond addition of new replication origins, antibiotic resistance markers and version numbers be needed.

**Table 2:** Numeric abbreviations for plasmid replication origins in BioBrick vector nomenclature.

*Number*	*Replication origin*	*Copy number*	*Purpose*
1	modified pMB1 derived from pUC19	500–700	Easy plasmid DNA purification
2	F and P1 lytic derived from pSCANS-1-BNL [67]	1–2 inducible to high copy	Inducible copy number
3	p15A derived from pMR101	10–12	Multi-plasmid engineered systems
4	rep101, repA derived from pSC101	5	Small cell to cell copy number variation
5	derived from F plasmid	1–2	Improved plasmid stability
6	pMB1 derived from pBR322	15–20	Multi-plasmid engineered systems

BioBrick vector names take the form pSB#X#. The first number indicates the identity of the origin of replication. The number, corresponding replication origin, expected plasmid copy number and typical purpose of that origin are listed [38]. To expand the list to include additional replication origins, document additions at the Registry of Standard Biological Parts [66].

**Table 3:** Letter abbreviations for antibiotic resistance markers in BioBrick vector nomenclature.

*Code*	*Antibiotic*
A	ampicillin
C	chloramphenicol
E	erythromycin
G	gentamycin
K	kanamycin
N	neomycin
Na	nalidixic acid
R	rifampicin
S	spectinomycin
St	streptomycin
T	tetracycline
Tm	trimethoprim
Z	zeocin

BioBrick vector names take the form pSB#X#. The letter X indicates the antibiotic to which the vector confers resistance. The letter code and corresponding antibiotic resistance marker are listed. The absence of a letter indicates that no antibiotic is present. Multiple resistance markers in a vector are indicated by successive codes in alphabetical order e.g., AK, StT, AC and AKT. To expand the list to include additional antibiotic resistance markers, document additions at the Registry of Standard Biological Parts [66].

**Table 4:** Numeric abbreviations for vector version number in BioBrick vector nomenclature.

*Number*	*Key features*	*Purpose*	*Example*	*Designer*
0	absent or incomplete BioBrick cloning site		pSB2K0	Brookhaven National Lab
1	complete BioBrick cloning site (BCS)	assembly of BioBrick parts	pSB4A1	Reshma Shetty
2	5' terminator and BCS	transcriptional insulation of vector upstream of cloned BioBrick part	pSB1A2	Tom Knight
3	5' terminator and BCS and 3' terminator	transcriptional insulation of vector downstream of cloned BioBrick part	pSB1AC3	Reshma Shetty & Tom Knight
4	pSB2K3-derived vector free of many restriction sites	Genome refactoring [68]	pSB2K4	Leon Chan
5	constructed from BioBrick base vector	standardized BioBrick vector design	pSB4K5	Reshma Shetty
6	Reserved	-	-	-
7	BCS flanked by terminator BBa_B0015	transcriptional insulation of cloned BioBrick part	pSB1A7	Karmella Haynes
8	Unassigned	-	-	-
9	Unassigned	-	-	-
10	Screening plasmid v1.0 [69]	characterization of single input, single output transcriptional devices	pSB1A10	Josh Michener & Jason Kelly

BioBrick vector names take the form pSB#X#. The second number indicates the BioBrick vector version number. The version number, key features, purpose for which that version was designed, example vector, and vector designer(s) are listed. To expand the list to include new vector version numbers, document additions at the Registry of Standard Biological Parts [66].

## Abbreviations

PCR – polymerase chain reaction. bp – base pairs. kb – kilobase (1000 base pairs).

## Competing interests

The author(s) declare that they have no competing interests.

## Authors' contributions

RS and TK designed the BioBrick standard biological parts and vectors described in this work. RS carried out all construction and testing. RS and DE wrote the manuscript. All authors read and approved the final manuscript.
